# O046. Color vision and visual cortex excitability are impaired in episodic migraine. Simply coexisting or pathophysiologically related dysfunctions?

**DOI:** 10.1186/1129-2377-16-S1-A57

**Published:** 2015-09-28

**Authors:** Filippo Brighina, Viviana Firpo, Simona Maccora, Vittoria Calabró, Fabio Lombardo, Giuseppe Cosentino, Roberta Baschi, Nadia Bolognini, Giuseppe Vallar, Brigida Fierro

**Affiliations:** Dipartimento di Biomedicine Sperimentali e Neuroscienze cliniche (BioNec), Università di Palermo, Palermo, Italy; Dipartimento di Psicologia, Università di Milano Bicocca, Milan, Italy

## Background and objectives

Evidence of abnormal color vision processing in migraine comes from observation of positive symptoms during visual aura, effects of strong color contrast triggering attacks and of colored-spectacles reducing migraine frequency. Although the central or peripheral basis of such color misperception remains unclear, several authors reported a selective deficit of shortwavelength cones (S-cones)[1]. Sound-induced flash illusions (SIFI) are a simple way to describe visual distorsion induced by acoustic perception. SIFI critically depend on excitability of primary visual cortex (V1) as they are reduced by facilitatory anodal transcranial direct current stimulation (tDCS) over V1 in healthy subjects[2]. We observed diminished SIFI in episodic migraine patients, especially in those with aura (MA) and during the attack[3] in agreement with the hypothesis of visual cortex hyperexcitability. Aim of the present study was to explore the potential correlation between cones dysfunction (evaluated by colorimetric scales) and visual cortex hyperexcitability (tested by SIFI) in episodic migraine without aura patients (MoA).

## Materials and methods

Twenty-two MoA patients (4 M; mean age 35.8±11.1 years) and 12 unimpaired healthy volunteers with no family history of migraine (9 M; mean age 27.7±13.2 years) were enrolled. Migraine patients were tested interictally. None of the patients enrolled had taken any prophylactic drug during the 3 months prior to the experiment.

Every participant took part in two randomized experimental sessions. Experiment 1 consisted of the Farnsworth-Munsell 100-hue test: the disks in 4 different trays had to be arranged to form a smooth color sequence between two reference disks. Experiment 2 consisted in reporting the number of flashes seen on a black screen in isolation or in different combinations with beeps.

## Results

Cone deficits were more prevalent in the migraine (72%) than in the control group (0.08%). S-cone deficit (56.2%) was still the most frequent alteration detected in our sample. Accordingly with previous results MoA patients as a whole showed no significant difference in SIFI perception as compared to controls even if a trend towards reduced illusory percept was found. However, when performing subgroup analyses, the MoA group with S-Cone defect showed significantly less fission illusions as compared to the group of healthy subjects (p < .0001).

## Discussion and conclusions

S-Cone dysfunction is highly prevalent in migraine; patients with such functional retinal impairment show higher levels of visual cortical excitability. The results of the study seem to establish a strong (causal?) relationship between retinal and visual cortical dysfunctions found in migraine. Further evidence (in migraine with aura and in the ictal phase) is needed to define the role and interplay of such abnormalities in migraine pathophysiology.

Written informed consent to publication was obtained from the patient(s).
